# Study on the colonization changes with different dark septate endophytes and their regulation of the growth and physiological mechanisms of *Pinus sylvestris* var. *mongolica* under drought stress

**DOI:** 10.7717/peerj.20720

**Published:** 2026-03-04

**Authors:** Peng Shao, Xun Deng, Siwen Zhong, Shixian Liao, Zheng Wang, Xiaoshuang Song

**Affiliations:** 1 Institute of Forestry Protection, Heilongjiang Forestry Academy, Harbin, China; 2 Karamay Forestry and Grassland Bureau, Karamay, China; 3 College of Forestry, Northeast Forestry University, Harbin, China

**Keywords:** Colonization morphology, Dark septate endophytes, Inoculation, *Pinus sylvestris* var. *mongolica*, Water-deficit stress

## Abstract

**Objective:**

This study aimed to understand the colonization effects of dark septate endophytes (DSE) on *Pinus sylvestris* var. *mongolica* under drought stress.

**Method:**

With the pot experiment in the greenhouse, the seedlings of *P. sylvestris* var. *mongolica* were inoculated with J09, J32, J35, A065, and the sterile PD medium. Under well-watered (WW) (80% field water capacity), moderate-watered (MW) (60% field water capacity), and low-watered (LW) (LW) conditions (40% field water capacity), the difference in the colonization morphology of DSE, the growth index of seedlings, physiological indexes of seedlings, physical and chemical properties of rhizosphere soil, and enzyme activities of rhizosphere soils were studied.

**Result:**

The effects of different DSE strains on drought tolerance of annual seedlings of *P. sylvestris* var. *mongolica* were determined by indoor weighing and water supplement methods. With the strength of drought, the diameter of hyphae became thicker, the septum became shorter, and the number of microsclerotia increased. The change of DSE colonization morphology may be one of the mechanisms to improve the drought tolerance of host plants, and the type of DSE colonization significantly affects the drought tolerance of host plants. Inoculation of J09 and A065 can alleviate the adverse effects of moderate and severe drought stress on host plants by improving plant growth indicators, increasing physiological indicators, enhancing rhizosphere soil physicochemical properties, and maintaining higher rhizosphere soil enzyme activity, respectively. Two-way analysis of variance (ANOVA) results indicated that the interaction between DSE and drought stress significantly affected the growth and physiological indexes of the plants, enzyme activities, and physicochemical properties of rhizosphere soil (*P* < 0.05).

**Conclusion:**

J09 and A065 have the potential to be applied in the microbial fertilizer for seeding in a drought area.

## Introduction

Mongolian pine (*Pinus. sylvestris* var. *Mongolia*), a geographical variant of *Scots pine* (*Pinus. sylvestris*), belongs to the pine family (Song et al., 2021). It is naturally distributed in the Heilongjiang and Inner Mongolia provinces of China and introduced in Zhanggutai, Liaoning provinces, and Yulin, Shanxi provinces, China for restoring degraded soil because of its rapid growth and drought resistance (Deng et al., 2020). *Pinus sylvestris* var. *Mongolia* has been selected as an experimental subject as it mainly functions in environmental restoration and ecological conservation (Song et al., 2021; Deng et al., 2020).

The frequency of drought is rising sharply and has become a crucial ecological factor impacting the distribution of humans and plants (Li et al., 2022). Drought inhibits root development, reduces the ability of plants to absorb minerals and water, and even causes plants to die (Li et al., 2022; Farooq et al., 2009). Using xerophytes to restore the ecosystem of deserts is considered a universally effective method in many nations and regions (He, Wang & Hou, 2019; De Vries et al., 2020). The colonization and growth of microorganisms depend on the delivery of photosynthates from association plants (Santos et al., 2021). Conversely, the natural habitats of plants often contain microorganisms that are crucial for promoting organic matter decomposition and nutrient transformation. In particular, some of these microbes help enhance plant tolerance to drought in arid areas (He, Wang & Hou, 2019). Therefore, alleviating drought stress and strengthening growth in host plants by associated fungal endophytes, such as dark septate endophytes (DSE), may be an efficient strategy (Santos et al., 2021).

DSE are significant root endophytes that exhibit broad distribution, especially in the roots of plants in extreme environments, including arid, swampy, mountainous, and high altitudes (Jumpponen, 2001). They are found in more than 600 plant species (Jumpponen & James, 1998), and one of their distinguishing characteristics is their dark septate hyphae and melanized microsclerotia (Liu, Zhang & Zhao, 2009). Most of the colonies cultured *in vitro* were black, gray, and brown, and the mycelia grew slowly, with most of them failing to produce spores (Mandyam & Jumpponen, 2005). Various DSE has been found and isolated from grasses, shrubs, and trees in arid regions by many scientists (Jumpponen & James, 1998). For instance, Barrow (2003) investigated DSE in *Bouteloua* sp. from the arid southwestern USA region and discovered the typical characteristics of septate hyphae and microsclerotia within plant roots. DSE was observed and isolated in the root of *Ammopiptanthus mongolicus* (Li et al., 2018), *Hedysarum scoparium* (Li et al., 2019), and *P*. *sylvestris* var. *mongolica* (Deng et al., 2020) in northern China. The prevalence of these environments indicates their important ecological function. The DSE facilitate the growth of their host plants by increasing their absorption of nitrogen (N), phosphorus (P), and potassium (K) mineral nutrients, improving the rhizosphere environment, and enhancing plant photosynthesis (He et al., 2022a; He et al., 2022b; He et al., 2022c; Berthelot, Blaudez & Leyval, 2017). DSE induces plant disease resistance by colonising the host plants (Newsham, 2011). For example, Surono (2018) found that inoculated DSE could prevent the invasion of pathogens effectively. The DSE A024 by screening has a significant inhibitory effect on *Rhizoctonia solani* (He et al., 2022a; He et al., 2022b; He et al., 2022c). Research has demonstrated that DSE enhance plants’ tolerance to salt-alkali and heavy metal stress through multiple mechanisms: promoting host plant growth, facilitating water and nutrient uptake, boosting the resistance of hosts to oxidative stress, and secreting secondary metabolites (Narisawa, Usuki & Hashiba, 2004; Narisawa, Hambleton & Currah, 2007; Liu et al., 2021).

Although it has been widely demonstrated that DSE can enhance the survival of host plants in arid environments (Mandyam & Jumpponen, 2005), studies have primarily focused on the distribution, isolation, and identification of DSE strains (Zhang et al., 2021; Mandyam & Jumpponen, 2005). For instance, Han et al. (2021) found that DSE colonization and diversity showed significant spatiotemporal heterogeneity and were closely related to soil factors in the roots of *Lycium ruthenicum* Murr in the desert region of northwest China. He, Wang & Hou (2020) reported that *Glycyrrhiza uralensis* was highly infected by DSE in different arid areas and had species diversity. Moreover, some studies have focused on inoculation with DSE under well-watered and drought to evaluate the effects of DSE on the drought tolerance of host plants. For example, He et al. (2022a), He et al. (2022b) and He et al. (2022c) found that inoculated DSE could promote the growth of *L. ruthenicum* under well-watered and drought stress. In another study, He, Wang & Hou (2019) found that inoculation with *Acrocalymma vagum and Paraboeremia putaminum* increased the biomass and glycyrrhizin content of licorice plants under well-watered and drought stress treatments. Even though the direct effects of DSE inoculants on plant growth and physiological indexes have been reported widely, Zhu et al. (2015) reported that the inoculation of *Leptodontidium* sp. strain significantly enhanced growth parameters as well as improved the total flavonoid and icariin content. Li et al. (2019) investigated the effect of DSE inoculated on *H. scoparium* under water shortage stress by increasing biomass, nutrient concentration, and antioxidant enzymatic activity, but there was little information on the contribution of DSE inoculated on host plant growth parameters, nutrient indices, rhizosphere soil enzymatic activity and colonization structure; hence we used weighing water replenishing method to simulate drought stress by setting up three concentrations to explore drought tolerance mechanism. Therefore, this study aimed to (1) observe the structure of DSE under different drought stress, (2) evaluate the effect of different drought stress on DSE colonization, (3) determine the effect on growth parameters, nutrient index, and enzyme activity of inoculation with DSE strains, (4) assess the effect of DSE inoculation on soil nutrient index and enzyme activity, and (5) finally explore the use of different DSE in promoting the growth of seedings, under drought stress.

## Material and Methods

### Fungal isolates and plant materials

In this study, four DSE fungi were isolated from the roots of *P. sylvestris* var. *mongolica*, which naturally grows in the Jiagedaqi District of Heilongjiang Province, Northeast China. These fungi can tolerate drought under *in vitro* screening experiments. They were identified *via* morphology and internal transcribed spacer (ITS) phylogeny previously (Li et al., 2018), including *Phialocephala* sp. (J09), *Phialocephala fortinii* (J32), *Gaeumannomyces caricis* (J35), and *Stagonospora bicolor* (A065). The strains were preserved at 4 °C in the Forest Microbiology Laboratory of Heilongjiang Forest Protection Institute.

These four DSE strains were grown on Petri dishes with potato dextrose agar (PDA) medium at PH 6.0. Inoculums of fungi suspension were obtained by transferring the seven mm plugs to a liquid PDA medium separately cultured under agitation (150 rpm) in the dark at 25 °C after seven days (Deng et al., 2020).

### Design of experiments and inoculation of seedlings

The experiment (five inoculation treatments and three water treatments) was conducted in the greenhouse laboratory of the Heilongjiang Forest Protection Research Institute and repeated 6 times (Li et al., 2019). The inoculation treatments included inoculation with J09, J32, J35, A065, and a non-inoculated control (CK). Three levels of water stress were tested: well-watered (WW), moderate-watered (MW), and low-watered (LW).

The seeds of *P. sylvestris* var. *mongolica* were sterilized with 0.5% potassium permanganate for 1 h and then rinsed several times with sterile water (Halifax et al., 2019). They were then planted under aseptic conditions on wet gauze in Petri dishes and left to germinate for three days at 25 °C (Deng et al., 2020). After germination, seeds were transferred to plastic pots (15 cm ×15 cm, 30 seeds per plot) (Song et al., 2021). The soil mixture (peat soil/vermiculite/sand (2:1:1, v/v/v)) was autoclaved at 121 °C for 120 min, as was all water during the experimental process (Halifax et al., 2019). All pots were kept in the greenhouse with 14 h light/10 h dark photoperiod, a temperature of 30 °C/22 °C (day/night) with 60% average relative humidity (Zuo et al., 2020). Every pot was watered once every two days and poured on once a week with Hoagland nutrient solution for 15 days. Using a sterile punch, cut two five mm diameter pieces of the strain cake. Inoculate these pieces into 150 ml of PD medium and shake the mixture for 14 days at 28 °C and 170 rpm for a fungal solution. The fungal inoculum was then applied to the seedlings three times (Deng et al., 2020; Halifax et al., 2019).

Following a month, one-third of the plant were subjected to WW treatment (80% field water capacity), one-third of the seedings were subjected to MW treatment (60% field water capacity), and the remaining one-third were subjected to LW treatment (40% field water capacity) (He et al., 2022a; He et al., 2022b; He et al., 2022c). Water loss was replenished with sterile water to maintain the desired field capacity by daily weighing between 9:00–11:00 a.m. (Li et al., 2019).

### Sampling and physiological parameter analysis of the seedlings

The roots and stems of the seedlings were collected from the soil by careful washing with tap water (Zuo et al., 2020; Torres-Júnior et al., 2018). The physiological indices of the seedlings included the height, the fresh weight, the dry weight, and the diameter of the shoot. Determine the dry biomass by drying it to a constant weight in an oven at a temperature of 85 °C (Torres-Júnior et al., 2018).

### Microscopic observation of root colonization

The ink-vinegar staining method was used to determine the infection rate of dark septate endophytic fungi in root samples (Yang et al., 2010). The roots were fixed with formaldehyde-acetic acid-ethanol (FAA) solution for 24 h, cleared with 10% KOH at 90 °C for 90 min, then bleached with 30% H_2_O_2_ for 5 min, acidified with lactic acid with 5 min, and dyed with 5% ink-vinegar for 5 min. Root samples were then soaked in sterile water for 12 h and examined under a microscope (Wu et al., 2020). For each treatment, 20 random roots of 0.5 cm in length were selected.

Colonization rate of DSE (%) = (length of colonization roots/total length of roots) ×100%.

### Determination of plant enzyme activities

Superoxide dismutase (SOD), catalase (CAT), peroxidase (POD), malondialdehyde (MDA), plant soluble sugars (PSS), and proline (PRO) were measured with a kit from Nanjing Jian Cheng Bioengineering Company (Deng et al., 2020).

### Determination of soil enzyme activities and physicochemical properties

Rhizosphere soil was collected and sieved through a 20-mesh sieve after harvesting the plants. For the determination of rhizosphere soil enzyme activity (Song et al., 2021), a total of 10 g of rhizosphere soil from each treatment group was weighed and stored at 4 °C. The remaining rhizosphere soil was air-dried and further used to measure its physicochemical properties. Total nitrogen (TN) was measured using the approach of Kjeldahl (Halifax et al., 2019). Total phosphorus (TP) was measured using the Mo-Sb anti-colorimetry method (Deng et al., 2020), and flame photometry was used to measure total potassium (TK). Organic matter (OM) was measured using the potassium dichromate oxidation external heating method (Deng et al., 2020). Available nitrogen (AN) was measured by the alkaline hydrolysis diffusion method. Available phosphorus (AP) was measured using the sodium bicarbonate extraction method (Song et al., 2021), while available potassium (AK) was measured by the NH_4_OAc leaching flame photometry (Song et al., 2021). The activities of soil catalase (S-CAT), sucrase (S-SC), and urease (S-UE) were determined using the kit of Nanjing Jian Cheng Biological Engineering Company (Deng et al., 2020).

### Data analyses

Graph Prism 9 software was used to conduct a two-way analysis of variance (ANOVA) to assess the impacts of DSE, drought stress, and their interaction on DSE colonization, plant biomass, morphological index, physiological index, and soil physicochemical parameters (Li et al., 2019). The significance of differences between samples was determined using Duncan’s test with SPSS 21.0 (Song et al., 2021).

## Results

### DSE root colonization

**Figure 1:**
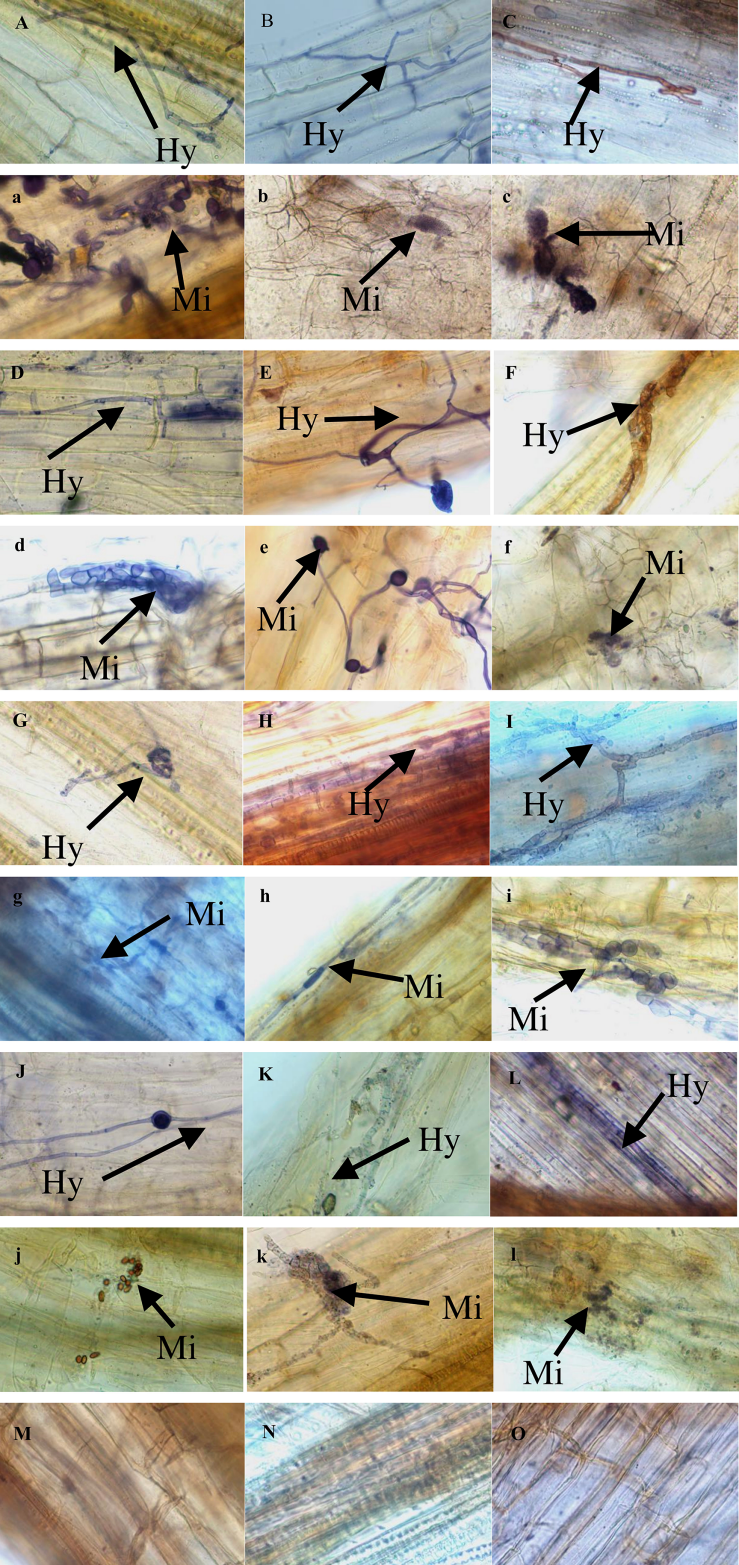
Colonization of four DSE strains in the roots of inoculated *P. sylvestris* var. *mongolica* plants after three months under different drought treatments. Note: The Hy indicates DSE hyphae; The Mi DSE microsclerotia (bars = 50 µm). (A, B, C) Roots of inoculated with J09 under WW, MW, and LW treatments; (D, E, F) roots of inoculated with J32 under WW, MW, and LW treatments; (G, H, I) roots of inoculated with J35 under WW, MW, and LW treatments; (J, K, L) roots of inoculated with A065 under WW, MW, and LW treatments; (M, N, O) roots of non-inoculated with DSE under WW, MW, and LW treatments.

**Figure 2:**
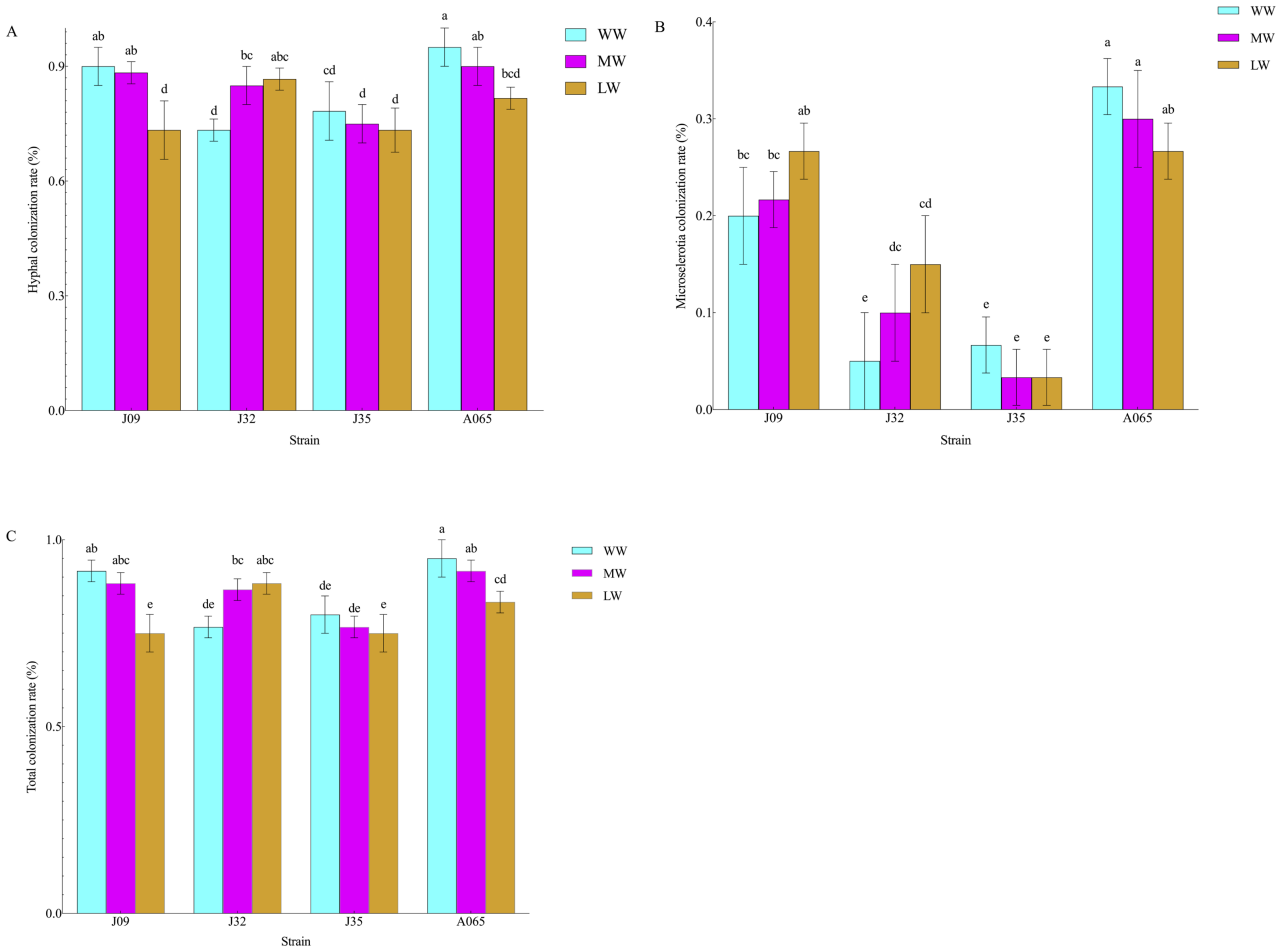
Colonization rates of DSE in the roots of *P. sylvestris* var. *mongolica*. Note: (A) hyphal colonization; (B) microsclerotial colonization; (C) total colonization. The lowercase letters indicate significant difference at *P* < 0.05 by Duncan’s multiple-range tests.

The morphology of DSE hyphal and microsclerotia were observed in all the inoculated root samples of the *P. sylvestris* var. *mongolica* plant (Fig. 1). On the other hand, the morphology of the DSE colonisation was not found in the control plants. Hyphal, microsclerotia, and total colonization were 90%, 20%, and 91.67% in the J09-inoculated plant, 73.33%, 5%, and 76.67% in the J32-inoculated plant, 78.33%, 6.67%, and 80% in J35-inoculated plant, and 95%, 33.33%, and 95% in A065-inoculated plant under WW conditions. Hyphal, microsclerotia, and total colonization were 88.33%, 21.67%, and 88.33% in the J09-inoculated plant, 85%, 10%, and 86.67% in the J32-inoculated plant, 75%, 3.33%, and 76.67% in J35-inoculated plant, and 90%, 30%, and 91.67% in A065-inoculated plant under MW conditions. Hyphal, microsclerotia, and total colonization were 73.33%, 26.67%, and 75% in the J09-inoculated plant, 86.67%, 15%, and 88.33% in the J32-inoculated plant, 73.33%, 3.33%, and 75% in J35-inoculated plant, and 81.67%, 26.67%, and 83.33% in A065-inoculated plant under LW conditions (Fig. 2). With the strength of drought, the diameter of hyphae became thicker, and the septum became shorter (Fig. 1). Two-way ANOVA findings revealed that microsclerotia, and total colonization rate were significantly influenced by DSE and the total colonization rate was affected by the interaction between DSE and drought stress (*P* < 0.05) (Table 1). The microsclerotia colonization increased with the strength of drought in J09 and J32-inoculated plants. However, hyphal and total colonization decreased with drought besides the J32-inoculated plant.

### Plant morphological and biomass parameters

**Figure 3:**
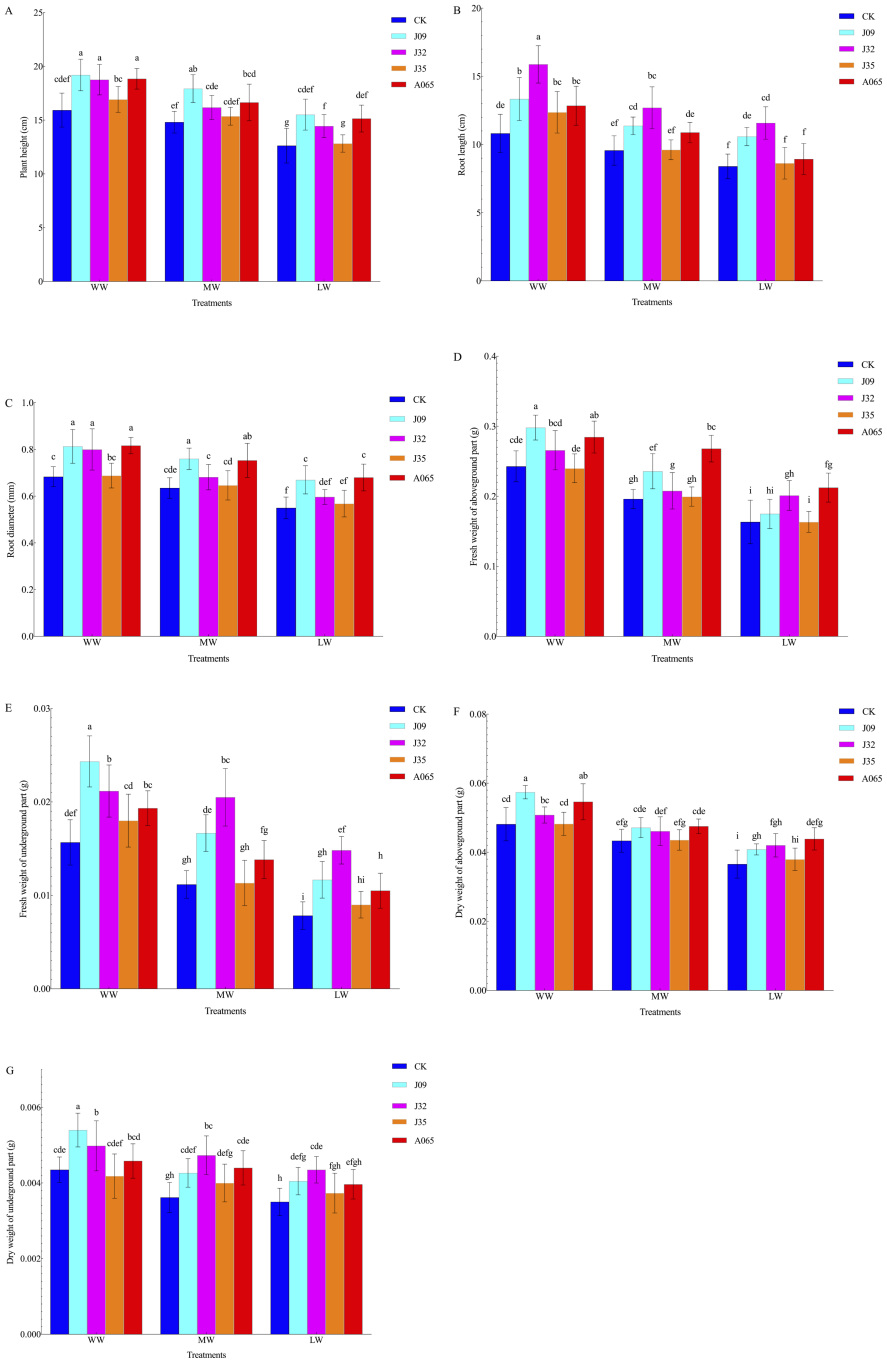
Effect of different DSE on morphological parameters of growth of *P. sylvestris* var. *mongolica* seedlings. Note: (A) Plant height of seeding; (B) root length of seeding; (C) root diameter of seeding; (D) fresh weight of aboveground part of seeding; (E) fresh weight of the underground part of seeding (F) dry weight of aboveground part of seeding (G) dry weigh of underground part of seeding. Each histogram represents the mean SD of four independent experiments. Different letters on columns indicate significant difference (*P* < 0.05).

Inoculation with DSE influenced the morphological characteristics of the *P. sylvestris* var. *mongolica* seedlings (Fig. 3). Compared with CK, after inoculation with J09, J32, J35, and A065, the plant height increased by 20.53%, 17.77%, 6.28%, and 18.33% under WW conditions, 20.99%, 9.18%, 3.64%, and 12.35% under MW conditions, and 22.88%, 14.41%, 1.58%, and 19.95% LW conditions, respectively. Furthermore, the root length increased by 23.20%, 46.77%, 14.30%, and 18.48%, 18.81%, 32.71%, 0.52%, and 13.69%, and 25.95%, 37.86%, 2.74%, and 6.31%, respectively, while the root diameter increased by 19.12%, 17.65%, 0.73%, and 20.15%, 20.63%, 8.21%, 1.84%, and 18.63%, and 21.81%, 8.49%, 3.27%, and 23.64%, respectively. Inoculation with DSE influenced the biomass parameters of the *P. sylvestris* var. *mongolica* seedlings. The fresh and dry shoot biomass in the J09-inoculated plant increased the most, 22.82% and 13.40%, respectively, under WW conditions. The fresh and dry shoot biomass in A065-inoculated plants increased the most, 36.70% and 9.64%, and 29.97% and 20.01%, under MW and LW conditions, respectively. The fresh and dry root biomass in the J32-inoculated plant increased the most. The growth parameters and the biomass of the seedlings of *P. sylvestris* var. *mongolica* were significantly affected by the DSE and the drought stress (*P* < 0.05) (Table 2). At the same time, the fresh weight of the aboveground part was affected by the interaction between DSE and drought stress. The plant height and root diameter of J09 and A065 inoculated under drought stress were higher than that of other treatments, and it did not change after inoculation with J35. The root length of J32 inoculated under drought stress was higher than that of other treatments. The fresh and dry weight of the aboveground part after inoculation with J09 was higher compared to other treatments under WW treatments, while after inoculation with A065, the fresh and dry weight of the aboveground part was higher than that of other treatments under MW and LW treatments. The fresh and dry weight of the underground part after inoculation with J09 was higher than that of other treatments under WW treatments, and inoculation with J32 was higher than that of other treatments under MW and LW treatments.

**Table 1:** Two-way ANOVA of the effect of DSE and water condition on DSE colonization rate of *P. sylvestris* var. *mongolica*.

	DSE		Drought stress		DSE′ drought stress	
	*F*	*P*	*F*	*P*	*F*	*P*
Hyphal colonization	8.25	0.085	7.63	0.096	7.54	0.056
Microsclerotia colonization	171.0	0.004	1.60	0.332	1.96	0.265
Total colonization	19.50	0.047	19.90	0.035	11.0	0.024

### Enzyme activities of seedings

**Figure 4:**
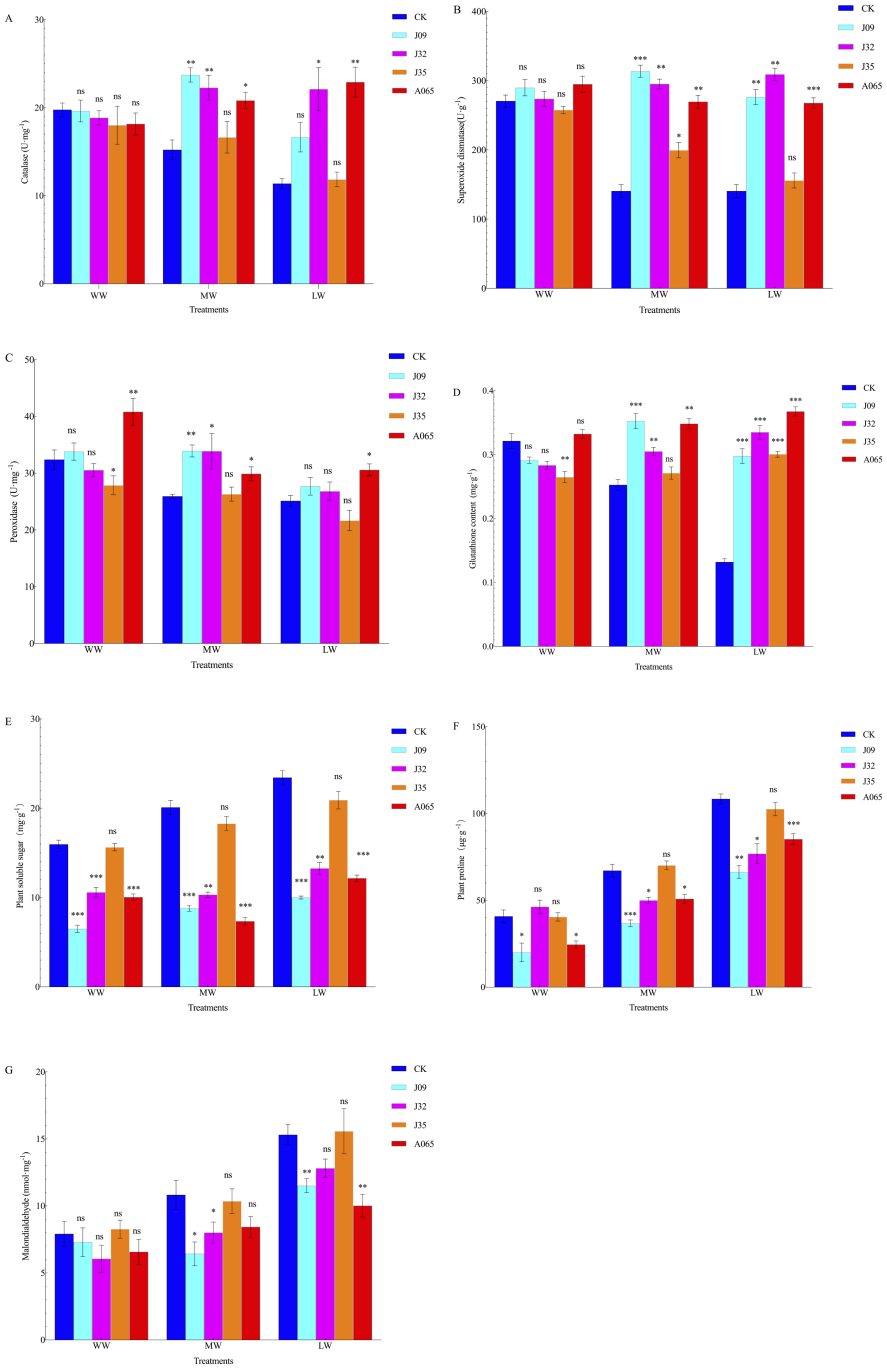
The enzyme activity indexes of seedlings after inoculation in different drought stress. Note: (A) CAT index of seeding; (B) SOD index of seeding; (C) POD index of seeding; (D) GSH index of seeding; (E) PSS (F) PRO index of seeding; (E) MDA index of seeding. Each histogram represents the mean SD of four independent experiments. Compared with CK, “*” means *P* < 0.05; “**” means *P* < 0.01; “***” means *P* < 0.005; “ns” means *P* > 0.05.

Under different drought conditions, inoculation with DSE had some effect on plant enzyme activity (Fig. 4). Compared with CK treatment, inoculation with J09, J32, J35, and A065 treatments significantly increased CAT by 55.91%, 46.35%, 9.36%, and 36.81% and 46.34%, 94.37%, 4.20%, and 101.41% in treatments of MW and LW, respectively. Inoculation with J09, J32, and A065 treatments significantly increased SOD activity by 39.53%, 31.33%, and 19.15% and 96.58%, 119.91%, and 90.59% under MW and LW treatments, respectively. Inoculation with J09, J32, and A065 resulted in the largest increase in POD activity by 30.87%, 30.64%, and 15.32% and 10.27%, 6.75%, and 21.78% under MW and LW treatments, respectively. However, inoculation with J35 treatment decreased SOD and POD activity in drought stress compared with CK; inoculation with J09 had the largest increase in CAT and SOD activities under MW treatment, while inoculation with A065 had the largest increase in POD activity under WW treatment. Under MW and LW treatments, after inoculation, J09, J32, J35, and A065 had increased GSH content by 39.55%, 20.70%, 19.01%, and 37.89% and 125.78%, 153.90%, 127.89%, and 178.62%. Under WW, MW, and LW treatments, after inoculation, J09, J32, J35, and A065 PSS content reduced by 145.67%, 50.80%, 2.11%, and 58.85%, 128.40%, 94.77%, 9.94%, and 173.50%, and 134.28%, 76.59%, 12.05%, and 92.60%, respectively. PRO content was 82.42%, 31.99%, and 34.35%, and 41.09%, 63.48%, 27.23% lower in J09, J32, and A065 inoculation, while the MDA content was 68.10%, 35.13%, and 28.36%, and 33.06%, 19.52%, 52.90% lower in J09, J32, and A065 inoculation than in CK under WW and LW treatments, respectively. While PRO and MDA contents of the J35-inoculation were significantly higher than in CK under drought stress. The results of two-way ANOVA demonstrated that DSE and drought stress had effects on the contents of SOD, CAT, POD, PRO, PSS, GSH, and MDA and the interaction between DSE and drought stress had a significant impact on the contents of SOD, CAT, POD, PRO, and PSS (*P* < 0.05) (Table 3). The change in activities of SOD and POD first increased and then decreased with the strength of drought after inoculation with J09 and the same trend in CAT activity of inoculation with J32.

### Physicochemical properties and enzyme activity of rhizosphere soil

Inoculation with DSE increased soil nutrient content (Tables 4 and 5). Compared with CK, inoculation with J09 increased soil OM, TN, and AN content the most, 10.41%, 14.45%, and 21.99%, 25.71%, 37.50%, and 81.49%, and 64.56%, 106.01%, and 88.34% under WW, MW, and LW treatments, respectively. Inoculation with J32 increased soil TP and AP contents the most, 57.33%, 60.22%, and 58.40% and 135.80%, 112.42%, and 157.50% under different drought stress. TK content increased the most, 17.03% and 30.21%, with J09-inoculated soil under WW and LW treatments and 19.11% with the A065- inoculated soil under MW treatment. Inoculation with A065 increased soil AK content the most, 58.30%, under WW treatment. Inoculation with J09 significantly increased by 65.99% and 54.85% under MW and LW treatments.

**Figure 5:**
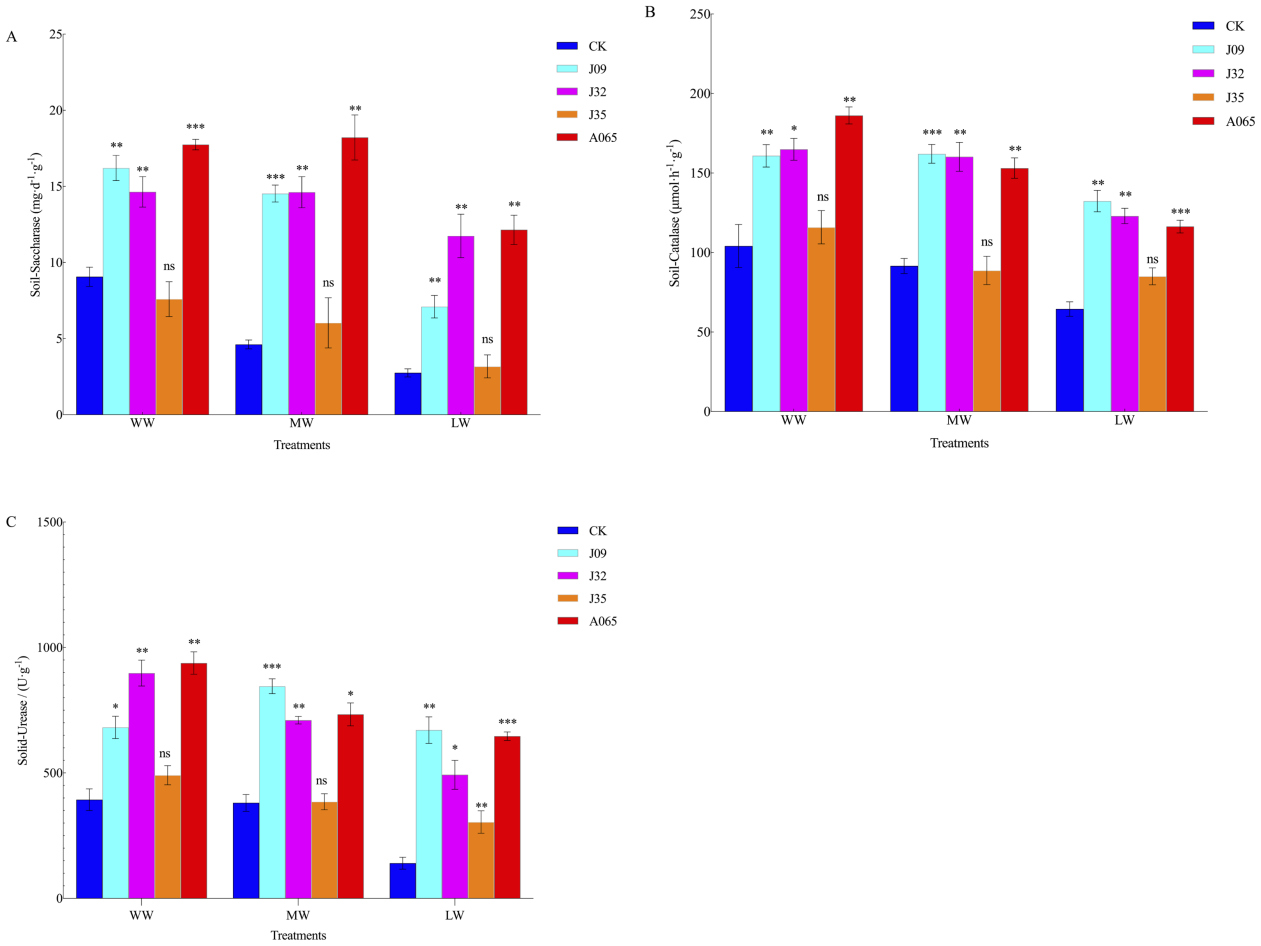
Effects of different dark septate endophyte (DSE) on enzyme activity of rhizosphere soil of *P. sylvestris* var. *mongolica* seedlings. Note: (A) S-SC activity of rhizosphere soil; (B) S-CAT activity of rhizosphere soil; (C) S-UE activity of rhizosphere soil. Each histogram represents the mean SD of four independent experiments. “*” means *P* < 0.05; “**” means *P* < 0.01; “***” means *P* < 0.005; “ns” means *P* > 0.05, compared with CK.

DSE inoculation had a certain effect on soil enzyme activity (Fig. 5, Table 6). Inoculation with J09, J32, and A065 significantly increased S-SC activity, increasing by 78.84%, 61.60%, and 95.81%, 214.45%, 216.45%, and 294.26%, and 158.18%, 326.91%, and 341.73%, respectively under different drought stress; S-CAT activity was 54.45%, 58.41%, and 78.84%, 76.96%, 74.94%, and 67.16%, and 105.29%, 90.77%, and 80.46% higher inoculation of J09, J32, and A065 than CK in WW, MW and LW treatments, respectively; S-UE activity was 73.25%, 128.29%, 24.86%, and 138.45%, 122.17%, 86.60%, 1.24%, and 92.63%, and 377.50%, 250.68%, 116.66%, and 360.31% higher inoculation of J09, J32, J35, and A065 than CK in WW, MW, and LW treatments, respectively. However, the J35-inoculation plant had no effect on the S-SC and S-CAT activities under drought stress.

**Table 2:** Two-way ANOVA of the effect of DSE and water condition on plant growth parameters of *P. sylvestris* var. *mongolica*.

	DSE		Drought stress		DSE′ drought stress	
	*F*	*P*	*F*	*P*	*F*	*P*
Plant height	20.60	<0.001	51.90	<0.001	0.43	0.762
Root diameter	15.40	<0.001	78.70	<0.001	0.66	0.590
Root length	57.20	<0.001	34.50	<0.001	1.15	0.359
Fresh weight of aboveground part	19.30	<0.001	170.0	<0.001	3.48	0.040
Fresh weight of underground part	33.0	<0.001	171.0	<0.001	3.09	0.055
Dry weight of aboveground part	12.40	<0.001	145.0	<0.001	1.35	0.297
Dry weight of underground part	10.90	0.002	35.0	<0.001	1.56	0.239

## Discussion

DSE’s ability to promote plant growth, control plant pathogens, and increase crop yields, along with their ability to colonize roots and perform specific microbial functions, make them potentially important biological control agents (Newsham, 2011). The *Phialocephala fortinii* s.l.—*Acephala applanata* (PAC) species complex has been reported as the most representative DSE in natural forest ecosystems in the Northern Hemisphere (Grunig et al., 2008). The four species of DSE used in this study were *Phialocephala* sp., *P. fortinii*, *Gaeumannomyces caricis ,* and *Stagonospora bicolor.* Several studies have been conducted on similar DSE strains; Jumpponen, Mattson & Trappe (1998) found that inoculation with *P*. *fortinii* significantly enhanced P and N uptake and pine growth, Deng et al. (2020) discovered *P. bamuru* could enhance the damping-off biocontrol and plant growth, and Ban et al. (2017) found that *Gaeumannomyces cylindrosporus* improved Pb tolerance of maize. DSE can form a complex symbiotic relationship with plants that can be neutral, facilitative, or inhibitory (Shinozaki & Yamaguchi-Shinozaki, 2007; Azad & Kaminskyj, 2016; Van der Heijden et al., 2015; Stoyke & Currah, 1991). In this study, inoculated DSE successfully colonized *P. sylvestris* var. *mongolica* roots, forming typical hyphae and microsclerotia (Fig. 1). Furthermore, host plant adaptation to colonization under drought stress was dependent on DSE species and water stress, with changes in drought leading to thicker hyphal diameter and shorter septum (Fig. 1). The hyphal and total colonization rate of inoculation with J09, J35, and A065 decreased with the strength of drought. In contrast, the hyphal and total colonization rate of inoculation with J32 and the DSE colonization rate of microsclerotia significantly increased, consistent with previous studies (Fig. 2) (He et al., 2022a; He et al., 2022b; He et al., 2022c). Host plant growth and stress resistance were improved by inoculation with DSE. Different DSE species enhance host plant performance in different ways (Santos et al., 2021). As per the study of Zhu et al. (2015), the plant height, root length, and shoot and biomass of *Epimedium wushanense* improved with the inoculation of *Leptodontidium* sp. Growth parameters and biomass of *P. sylvestris* var. *mongolica* increased with *P. bamuru* inoculation according to the study by Deng et al. (2020). In this study, all plants inoculated with DSE showed no adverse effects under water deficit conditions (Figs. 2; 3). The results of the two-way ANOVA showed that DSE had a significant effect on the morphological and biomass parameters of *P. sylvestris* var. *mongolica* (Table 2). Compared with non-inoculation treatments, the height, root length, root diameter, and biomass of the shoot and root of the seedings increased significantly after inoculation with DSE. Li et al. (2018) found that some DSE strains decreased plant biomass, while only three species promoted the growth of the host plant when nine DSE strains were inoculated into *A. mongolicus* (Fig. 3). This suggests that the increase in plant growth parameters of *P. sylvestris* var. *mongolica* may be mainly due to the altered DSE hyphal structure and increased microsclerotia under water deficit stress. Further evidence illustrates this point: among the four DSE strains, A065 produced the highest microsclerotia colonization rate, and inoculation of strain A065 had the most significant effect on seedling growth parameters.

**Table 3:** Two-way ANOVA of the effect of DSE and water condition on plant physiological parameters of *P. sylvestris* var. *mongolica*.

	DSE		Drought stress		DSE′ drought stress	
	*F*	*P*	*F*	*P*	*F*	*P*
POD	44.70	<0.001	86.50	<0.001	15.70	0.007
SOD	277.0	<0.001	722.0	<0.001	58.80	<0.001
CAT	29.60	<0.001	13.90	0.024	31.60	<0.001
GSH	276.0	<0.001	17.40	0.013	170	<0.001
PSS	1,246.0	<0.001	342.0	<0.001	37.0	<0.001
PRO	190.0	<0.001	1761.0	<0.001	17.0	0.003
MDA	29.50	0.001	489.0	<0.001	5.41	0.074

**Table 4:** Effects of different DSE on soil physicochemical parameters of *P. sylvestris* var. *mongolica* seedlings. Data (means ± SD, *n* = 3) followed by different letters among treatments indicate significant differences between treatments (*P* < 0.05).

Drought stress	Treat- ments	OM (g/kg)	TN (g/kg)	AN (mg/kg)	TP (g/kg)	AP (mg/kg)	TK (g/kg)	AK (mg/kg)
WW	CK	121.12 ± 2.72cd	1.05 ± 0.07def	125.68 ± 8.03ef	1.80 ± 0.06e	185.93 ± 26.13de	8.65 ± 0.42de	80.48 ± 7.55def
	J09	133.73 ± 2.38a	1.32 ± 0.04a	206.82 ± 8.11b	2.68 ± 0.09a	419.66 ± 40.84a	10.13 ± 0.31a	107.99 ± 8.50b
	J32	128.06 ± 1.98b	1.10 ± 0.04bcd	195.24 ± 6.11bc	2.83 ± 0.08ab	438.42 ± 45.83a	9.85 ± 0.08ab	98.93 ± 3.96bc
	J35	120.32 ± 1.99cd	0.99 ± 0.03f	161.73 ± 11.39d	1.88 ± 0.06de	210.96 ± 30.32d	8.99 ± 0.14cd	87.19 ± 6.37cde
	A065	129.20 ± 1.57ab	1.12 ± 0.03bc	197.84 ± 14.13bc	2.34 ± 0.08b	365.62 ± 28.34b	9.85 ± 0.12ab	127.38 ± 9.60a
MW	CK	112.49 ± 1.96ef	0.83 ± 0.06 h	109.63 ± 8.47fgh	1.52 ± 0.05gh	167.07 ± 20.41def	8.27 ± 0.24e	66.26 ± 4.53f
	J09	128.75 ± 1.43ab	1.14 ± 0.04b	225.85 ± 17.32a	2.38 ± 0.07b	320.07 ± 20.99bc	9.48 ± 0.33c	109.98 ± 7.66b
	J32	119.86 ± 4.06cd	1.03 ± 0.04def	156.60 ± 12.00d	2.43 ± 0.09b	354.89 ± 40.00b	9.35 ± 0.16c	92.93 ± 8.35cd
	J35	112.89 ± 3.35ef	0.87 ± 0.05gh	119.82 ± 10.56efg	1.62 ± 0.05fg	155.75 ± 19.43efg	8.29 ± 0.15e	78.98 ± 9.05def
	A065	125.38 ± 1.67bc	1.02 ± 0.03ef	186.97 ± 9.08c	1.75 ± 0.08ef	214.61 ± 32.44d	9.85 ± 0.11ab	92.62 ± 9.52cd
LW	CK	95.95 ± 2.08 g	0.60 ± 0.02j	99.18 ± 3.60 h	1.25 ± 0.08i	109.15 ± 13.92 g	7.04 ± 0.29 g	53.68 ± 4.59 h
	J09	117.05 ± 5.93de	1.08 ± 0.03bcd	186.79 ± 11.98c	2.13 ± 0.04c	274.56 ± 24.73c	9.17 ± 0.21ef	83.12 ± 7.13de
	J32	109.72 ± 4.22f	0.80 ± 0.06 h	136.19 ± 11.66e	1.98 ± 0.05cd	281.06 ± 33.17c	9.01 ± 0.18cd	80.64 ± 6.87bc
	J35	98.92 ± 1.90 g	0.71 ± 0.03i	103.63 ± 8.47gh	1.41 ± 0.24 h	116.61 ± 17.67fg	7.65 ± 0.58f	67.06 ± 7.03fg
	A065	118.08 ± 4.90de	0.90 ± 0.03 g	135.34 ± 11.92e	1.59 ± 0.05 g	152.03 ± 23.08efg	8.39 ± 0.19e	77.60 ± 10.66efg

**Table 5:** Two-way ANOVA of the effect of DSE and water condition on soil physicochemical parameters of *P. sylvestris* var. *mongolica*.

	DSE		Drought stress		DSE′ drought stress	
	*F*	*P*	*F*	*P*	*F*	*P*
OM	75.50	<0.001	95.60	0.010	2.59	0.214
TN	74.30	0.003	234.0	0.003	5.59	0.089
AN	102.0	0.007	205.0	0.003	4.99	0.093
TP	202.0	<0.001	244.0	0.004	3.27	0.177
AP	244.0	<0.001	286.0	0.001	31.90	0.008
TK	76.20	0.002	79.40	0.004	3.59	0.133
AK	32.0	0.012	135.0	0.006	6.34	0.106

**Table 6:** Two-way ANOVA of the effect of DSE and water condition on soil enzyme activity of *P. sylvestris* var. *mongolica*.

	DSE		Drought stress		DSE′ drought stress	
	*F*	*P*	*F*	*P*	*F*	*P*
S-SC	178.0	<0.001	279.0	<0.001	17.70	0.002
S-CAT	205.0	<0.001	255.0	<0.001	7.98	0.011
S-UE	379.0	<0.001	276.0	<0.001	17.60	0.002

Drought stress can adversely affect plants, resulting in the accumulation of reactive oxygen species (ROS) radicals and oxidative cell damage to plants, while plants can adjust the contest of antioxidants and osmotic potential to protect the plant from drought stress (Shinozaki & Yamaguchi-Shinozaki, 2007). Previous studies have demonstrated the protection system of antioxidant enzymes constituted by SOD, POD, and CAT that were formed in plant cells to eliminate or reduce the excess O^2−^ and ROS radicals produced by non-biological stress (such as drought stress) or biological stress (such as diseases) (Azad & Kaminskyj, 2016; Van der Heijden et al., 2015). It was found that DSE inoculation increased the activities of SOD, POD, and CAT, alleviating the adverse effects of drought stress on host plants. In the present study, host plants inoculated with J09, J32, and A065 contained significantly higher SOD, POD, and CAT concentrations than control plants under MW and LW treatments (Fig. 4). The change of activities of SOD and POD increased first and then decreased with the drought strength after inoculation with J09, and the same trend in CAT activity of inoculation with J32. These findings can be related to the study by Li et al. (2019), who found that DSE increased the tolerance of *H. scoparium* to water shortage stress by increasing antioxidant enzyme activity. GSH is also an antioxidant that can reduce cell damage and improve the survival of organisms in stressed habitats (Santos et al., 2021). The content of GSH in this study showed an increasing trend as drought stress increases, and inoculation treatments were higher than non-inoculation, suggesting that GSH may alleviate the drought stress on seedings. PRO and PSS are the primary osmoregulatory substances needed to maintain intracellular osmotic pressure stability (He et al., 2021a; He et al., 2021b; He et al., 2020; Silvana et al., 2017; Yakti et al., 2019). When plants are disturbed by external environmental conditions, they maintain the stability of the intracellular protoplast colloid by changing the content of PRO and PSS in the plant and avoid metabolic disorders or death of plants due to water loss. This study reported that the contents of PRO and PSS inoculation treatments were lower than CK treatment under drought stress, suggesting that DSE inoculation alleviates drought stress, increasing the host plant’s drought resistance capacity.

The soil environment is the substance in which plants survive, and differences in the soil environment have a direct effect on the growth and development of plants and the composition of microbial communities (Yakti et al., 2019; Lü, Zou & Wu, 2019; Lata et al., 2018). Soil enzymatic activity, soil organic matter, and available nitrogen are all increased by DSE inoculants (Chen et al., 2017; Zhang et al., 2017). The content of nitrogen, phosphorus, potassium, and other nutrients in the soil represents the potential fertility of the soil, according to Wang et al. (2015). Shen et al. (2020) found that nitrogen, phosphorus, and potassium levels in the rhizosphere of *Sinosenicio oldhamianus* treated with PGPR mixture were significantly higher than in the untreated control group. Jumpponen, Mattson & Trappe (1998) found that DSE colonized the roots of plants growing in a low nitrogen and low organic matter stress environment and promoted plant growth by increasing the content of soil fertility in the rhizosphere of plants. The DSE *Acrocalymma vague* and *Paraboeremia Putaminum* and *Trichoderma viride* have been co-inoculated with *Astragalus mongolicus* to improve the drought resistance of the host plant by increasing the levels of organic matter, available nitrogen, available phosphorus, and available potassium in the rhizosphere (He et al., 2022a; He et al., 2022b; He et al., 2022c). He et al. (2021a) and He et al. (2021b) found that inoculation with DSE improved the growth and drought tolerance of licorice under drought stress by altering the osmotic stress tolerance of the rhizosphere soil or by increasing soil nutrient solubility. The results of this experiment indicate that after inoculation with DSE other than J35, the contents of organic matter, total nitrogen, available nitrogen, total phosphorus, available phosphorus, total potassium, and available potassium in the rhizosphere soil of *P. sylvestris* var. *mongolica* significantly increased (Table 4), which is consistent with the research results of He et al. (2021a) and He et al. (2021b) on licorice inoculated with DSE. Soil urease can convert soil nitrogen from the organic state to the available state, which is closely related to the level of available nitrogen in the soil (Igalavithana et al., 2018; Kuscu, 2019). Soil catalase can hydrolyze hydrogen peroxide, reduce the toxicity of hydrogen peroxide to plant roots, and characterize soil organic matter content (Yu et al., 2019). Soil sucrase is closely related to soil microbial activity (Yang & Wang, 2002). In this study, under drought stress, the activities of S-CAT, S-SC, and S-UE in the rhizosphere soil of *P. sylvestris* var. *mongolica* inoculated with DSE, except J35, were better than those of the uninoculated control group, similar to previous research results. In our experiment, the seedling height, root length, root diameter, and biomass of seedlings after inoculation with DSE were significantly higher than those of the non-inoculated treatment, and the physicochemical properties and enzyme activity content of the rhizosphere soil were also significantly higher than those of the non-inoculated control group. Therefore, inoculation of DSE may promote plant growth by improving rhizosphere soil conditions and thus host plant drought resistance.

## Conclusions

In this study, we found that the DSE of experiments can effectively colonize the roots of *P. sylvestris* var. *mongolica* and increase drought resistance of the seedings by enhancing the growth parameters in addition to J35. These beneficial roles may be associated with the changes in soil physicochemical properties and plant enzyme activities of *P. sylvestris* var. *mongolica* with DSE inoculation. Especially under drought stress, the DSE of experiments showed thicker DSE hyphae, shorter septa, and significantly higher rates of microsclerotia colonization. This suggests that the change in DSE colonization morphology may be due to the drought tolerance mechanism of the host plant after inoculation with DSE. *P. sylvestris* var. *mongolica* plays a crucial role in environmental conservation; at the same time, DSE forms a symbiotic relationship with plants, and improving the rhizosphere microenvironment helps host plants to better adapt to adverse environmental pressures.This experiment showed that DSE J09, J32, J35, and A065 supported the drought tolerance of *P. sylvestris* var. *mongolica* to different degrees. Thus, the type of DSE has a significant effect on the drought tolerance of the host plant. Inoculation of J09 and A065 can alleviate the adverse effects of moderate and severe drought stress on host plants by improving plant growth indicators, increasing physiological indicators, enhancing rhizosphere soil physicochemical properties, and maintaining higher rhizosphere soil enzyme activity, respectively. Therefore, J09 and A065 have the potential to be applied in the microbial fertilizer of *P. sylvestris* var. *mongolica* in a drought area. The present study, which was only conducted in potted conditions and did not include field trials in the forest, also has certain limitations. What is the molecular mechanism by which DSE interacts with plants? These are issues that are the subject of further research. In this study, only DSE was used as a representative, however, soil micro-organisms often interact with each other to form plant rhizosphere microbial communities, and the next study can enrich the species of strains and increase forest experiment. In addition to the above biochemical effects, will the vaccination method affect the result? An important direction for future technological applications will be how to prepare mycorrhizal fertilizers that can be produced in large quantities.

##  Supplemental Information

10.7717/peerj.20720/supp-1 Supplemental Information 1 Colonization rates of DSE in the roots of *P. sylvestris* var. *mongolica*

10.7717/peerj.20720/supp-2 Supplemental Information 2 Effect of different DSE on morphological parameters of growth of *P. sylvestris* var. *mongolica* seedlings

10.7717/peerj.20720/supp-3 Supplemental Information 3 The enzyme activity indexes of seedlings after inoculation in different drought stress

10.7717/peerj.20720/supp-4 Supplemental Information 4 Effects of different DSE on soil physicochemical parameters of *P. sylvestris* var. *mongolica* seedlings

10.7717/peerj.20720/supp-5 Supplemental Information 5 Two-way ANOVA of the effect of DSE and water condition on soil physicochemical parameters of *P. sylvestris* var. *mongolica*

## Additional Information and Declarations

### Data Availability

The following information was supplied regarding data availability: The raw measurements are available in the Supplementary Files.
